# IL-1β Damages Fibrocartilage and Upregulates MMP-13 Expression in Fibrochondrocytes in the Condyle of the Temporomandibular Joint

**DOI:** 10.3390/ijms20092260

**Published:** 2019-05-07

**Authors:** Hessam Tabeian, Beatriz F. Betti, Cinthya dos Santos Cirqueira, Teun J. de Vries, Frank Lobbezoo, Anouk V. ter Linde, Behrouz Zandieh-Doulabi, Marije I. Koenders, Vincent Everts, Astrid D. Bakker

**Affiliations:** 1 Oral Cell Biology, Academic Centre for Dentistry Amsterdam, University of Amsterdam and Vrije Universiteit Amsterdam, 1081 LA Amsterdam, The Netherlands; h.tabeian@acta.nl (H.T.); b.f.betti@acta.nl (B.F.B.); anouk.terlinde@student.auc.nl (A.V.t.L.); b.zandiehdoulabi@acta.nl (B.Z.-D.); v.everts@acta.nl (V.E.); 2 Oral Kinesiology, Academic Centre for Dentistry Amsterdam, University of Amsterdam and Vrije Universiteit Amsterdam, 1081 LA Amsterdam, The Netherlands; f.lobbezoo@acta.nl; 3 Orthodontics, Academic Centre for Dentistry Amsterdam, University of Amsterdam and Vrije Universiteit Amsterdam, 1081 LA Amsterdam, The Netherlands; 4 Núcleo de Anatomia Patológica, Instituto Adolfo Lutz, São Paulo 01246-000, Brazil; cinthyaquiron@gmail.com; 5 Periodontology, Academic Centre for Dentistry Amsterdam, University of Amsterdam and Vrije Universiteit Amsterdam, 1081 LA Amsterdam, The Netherlands; teun.devries@acta.nl; 6 Rheumatology, Radboud University Medical Center, 6525 GA Nijmegen, The Netherlands; marije.koenders@radboudumc.nl

**Keywords:** ADAMTS4, ADAMTS5, fossa, cartilage degradation, arthritis, mechanical loading, MMP-13, IL1β, temporomandibular joint, juvenile idiopathic arthritis

## Abstract

The temporomandibular joint (TMJ), which differs anatomically and biochemically from hyaline cartilage-covered joints, is an under-recognized joint in arthritic disease, even though TMJ damage can have deleterious effects on physical appearance, pain and function. Here, we analyzed the effect of IL-1β, a cytokine highly expressed in arthritic joints, on TMJ fibrocartilage-derived cells, and we investigated the modulatory effect of mechanical loading on IL-1β-induced expression of catabolic enzymes. TMJ cartilage degradation was analyzed in 8–11-week-old mice deficient for IL-1 receptor antagonist (IL-1RA^−/−^) and wild-type controls. Cells were isolated from the juvenile porcine condyle, fossa, and disc, grown in agarose gels, and subjected to IL-1β (0.1–10 ng/mL) for 6 or 24 h. Expression of catabolic enzymes (ADAMTS and MMPs) was quantified by RT-qPCR and immunohistochemistry. Porcine condylar cells were stimulated with IL-1β for 12 h with IL-1β, followed by 8 h of 6% dynamic mechanical (tensile) strain, and gene expression of MMPs was quantified. Early signs of condylar cartilage damage were apparent in IL-1RA^−/−^ mice. In porcine cells, IL-1β strongly increased expression of the aggrecanases ADAMTS4 and ADAMTS5 by fibrochondrocytes from the fossa (13-fold and 7-fold) and enhanced the number of MMP-13 protein-expressing condylar cells (8-fold). Mechanical loading significantly lowered (3-fold) IL-1β-induced MMP-13 gene expression by condylar fibrochondrocytes. IL-1β induces TMJ condylar cartilage damage, possibly by enhancing MMP-13 production. Mechanical loading reduces IL-1β-induced MMP-13 gene expression, suggesting that mechanical stimuli may prevent cartilage damage of the TMJ in arthritic patients.

## 1. Introduction

The temporomandibular joint (TMJ) is a unique joint, consisting of a fossa, disc, and condyle that is essential for mastication, speech, and deglutition [1]. The major difference between the TMJ and other synovial joints is that the TMJ contains fibrocartilage rather than hyaline cartilage, i.e., it contains collagen type I in addition to collagen type II and proteoglycans [2]. More precisely, the matrix of all three anatomical structures of the TMJ contained collagen type I. The condyle and the fossa stained positive for collagen type II and proteoglycans, but the condyle contained considerably more collagen type II and proteoglycans than the fossa. The disc did not contain collagen type II, and the disc did not stain positive for proteoglycans [2]. The TMJ is an under-recognized joint in arthritic disease, while it is one of the most commonly affected joints in patients with juvenile idiopathic arthritis (JIA) [3]. It has been suggested that at the time of diagnosis, approximately 75% of JIA patients have problems with the TMJ [3]. JIA, the most prevalent type of arthritis of unknown cause in young children, is initiated before the age of 16 years old and is characterized by chronic inflammation of the joints, which can result in joint degradation. Affected children suffer from jaw pain but also jaw dysfunction, which can manifest in malocclusion [4] and a reduced maximum mouth opening [5]. How the cartilage of the TMJ is affected by inflammation in JIA and in other arthritic diseases with involvement of the TMJ remains elusive. 

One of the most potent inflammatory factors involved in hyaline cartilage degradation in many forms of arthritis is interleukin (IL)-1β [6]. This cytokine is responsible for hyaline cartilage matrix degradation by inducing expression of matrix metalloproteinases (MMPs) and disintegrin and metalloproteinase with thrombospondin motifs (ADAMTS) by chondrocytes [7,8]. The importance of IL-1β in the pathogenesis of systemic arthritic diseases is demonstrated by the success of treatment with IL-1 receptor antagonist (IL-1RA) [9]. However, it is unknown whether IL-1β also affects the integrity of the cartilaginous structures of the TMJ.

Since the TMJ is a secondary growth center, damage induced by catabolic factors during JIA can introduce growth abnormalities, resulting in asymmetric growth of the mandible [10] undersized jaw, and abnormal positioning of the maxilla [11]. Therefore, strategies to prevent TMJ joint damage, particularly in JIA patients, are highly desirable. Preferably, a non-invasive treatment should be deployed that inhibits the catabolic effect of inflammatory factors on TMJ cartilage. Mechanical loading of inflamed joints can be a promising approach towards achieving this. Moderate exercise has been shown to have a systematic anti-inflammatory effect by reducing the disease activity in rheumatoid arthritis (RA) patients [12]. Furthermore, mechanical loading reduced the expression of MMP-13 in synovial cells from RA patients [13]. However, it is not known whether mechanical loading will also reduce IL-1β-induced expression of catabolic factors in cells derived from the TMJ condyle, which is especially susceptible to damage in JIA [14].

We hypothesize that IL-1β plays an important role in inducing degradation of the TMJ cartilage, that it enhances expression of catabolic factors such as MMPs and ADAMTSs, and that mechanical stimuli can revert IL-1β-induced expression of catabolic factors. We have used different model systems to investigate this hypothesis. First of all, an IL1RA knock-out mouse model was used to investigate whether overactive IL-1β signaling induces histological signs of damage in the fibrocartilage tissue of the temporomandibular joint. The second and third part of the hypothesis was challenged using pig TMJ-derived cells. Pigs were chosen to isolate cells because they will yield more cells than mice and because the TMJ of this species is comparable with that of humans in cellular composition [15,16,17,18,19].

## 2. Results

### 2.1. IL-1βRA^−/−^ Mice Showed Early Signs of Condylar Cartilage Damage

**Figure 1:**
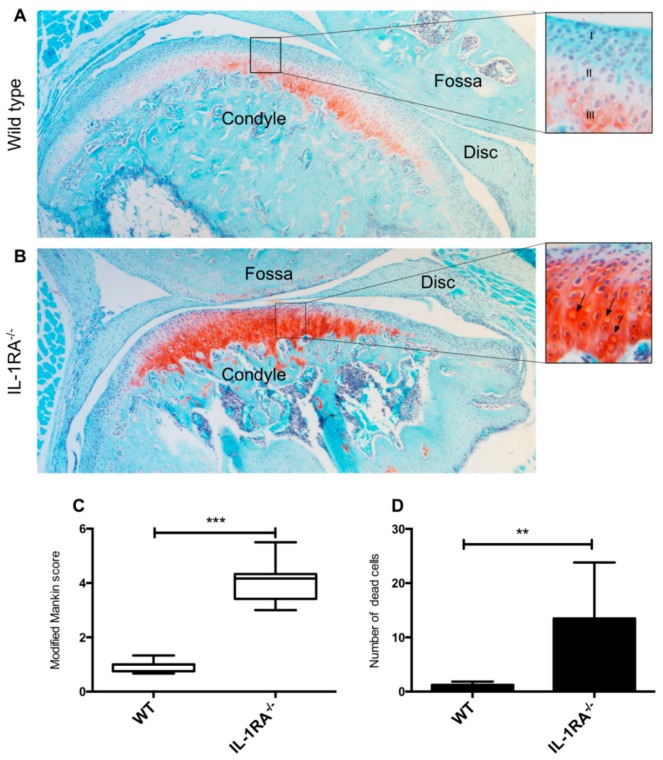
Histologic assessment of the temporomandibular joint (TMJ) of IL-1 receptor antagonist (IL-1RA^−/−^) and wild-type (WT) mice. Sagittal section of the condyles of IL-1RA^−/−^ and WT mice stained with Safranin O. (**A**) WT TMJ, original magnification 10×. The condyle cartilage can be divided into the fibrous, proliferative, and hypertrophic zones, indicated in the figure as I, II, III, respectively. In the WT sample the modest red staining is limited to zone III. (**B**) The IL-1R^−/−^ mice condyle showed a higher level of Safranin O staining in comparison to WT. In the IL-1R^−/−^ mice, Safranin O staining was not limited to the hypertrophic and the proliferative zone of the condyle but extended to the fibrous layer. Empty lacunae were frequently seen (arrows). (**C**) The Mankin score of the IL-1RA^−/−^ mice was higher than the WT. (**D**) The number of empty lacunae in the condyles of the IL-1RA^−/−^ mice was higher than in the WT. ** Significant difference between IL-1RA^−/−^ and WT mice, *p* < 0.01; *** significant difference between IL-1R^−/−^ and WT mice, *p* < 0.001, a *t*-test is used.

To investigate the role of IL-1β in TMJ damage, we assessed whether young mice that lack IL1-RA develop arthritis in the TMJ. Discs were barely visible in sections of mouse TMJ. Because of the similar histological appearance of fossa and disc tissue in both wild-type (WT) and IL-1RA^−/−^ mice, only the condyles were quantified. Safranin O staining was more intense in IL-1RA^−/−^ condyles compared to WT condyles (Figure 1A,B). In addition, the most superficial layer of the cartilage in IL-1RA^−/−^ condyles was positive for Safranin O staining (Figure 1B), which was not the case in WT mice (Figure 1A). The IL-1RA^−/−^ TMJ samples had a significantly higher Mankin score compared to the joints of the WT mice (*p* < 0.01) (Figure 1C). The IL-1RA^−/−^ condyles contained 11-fold more empty lacunae than the WT mice (*p* < 0.001) (Figure 1D).

### 2.2. Cells from the Fossa, Disc, and Condyle Expressed IL-1 Receptors

**Figure 2:**
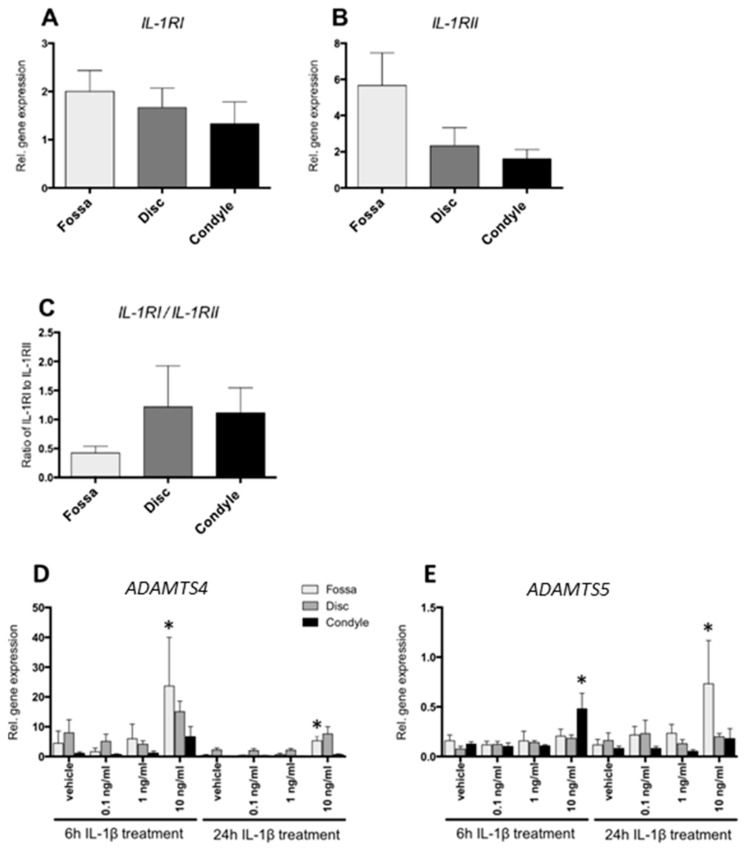
Relative gene expression of IL-1 receptor (IL-1R)I, IL-1RII, disintegrin and metalloproteinase with thrombospondin motifs (ADAMTS)4 and ADAMTS5 by porcine fossa, condyle and disc cells. (**A**) IL-1RI and (**B**) IL-1RII expression of the cells from fossa, disc, and condyle. All cells expressed IL-1RI and RII gene at similar levels. (**C**) The ratio between *IL-1RI* and *IL-1RII*. The ratio IL-1RI/IL-1RII was comparable for all cells. (**D**) *ADAMTS4* expression in the cells from the fossa, disc, and condyle. IL-1β incubation for 6 h enhanced ADAMTS4 expression in condyle cells. After 24 h of incubation with 10 ng/mL IL-1β, both fossa and discs showed an increase in ADAMTS4 expression in comparison to the vehicle-treated cells. (**E**) *ADAMTS5* expression in the cells from the fossa, disc, and condyle. Six hours of 10 ng/ml IL-1β treatment enhanced ADAMTS5 gene expression in condyle cells. After 24 h of 10 ng/mL IL-1β, the fossa cells showed an increased *ADAMTS5* expression. * Significant effect of treatment with IL-1β relative to vehicle, *p* < 0.05.

The ability of the cells isolated from porcine fossa, disc, and condyle cartilaginous structures to react to IL-1β was assessed by measuring gene expression of receptors for IL-1β. All cells from the three types of TMJ cartilage displayed similar gene expression levels for IL-1RI as well as of the mock receptor of IL-1β, IL-1RII (Figure 2A,B). The ratio of IL-1RI to IL-1RII gives a rough indication of the effectiveness of IL-1β to elicit downstream signaling. The three cartilaginous structures displayed similar IL-1RI/IL-1RII ratios (Figure 2C). Expression of IL-1RA and IL-1β was in most cases undetectable, and therefore no statistical analysis could be performed. 

### 2.3. IL-1β Increased ADAMTS4 and ADAMTS5 Gene Expression

IL-1β at 10 ng/mL enhanced ADAMTS4 gene expression by 5-fold after 6 h in cells from the fossa (*p* < 0.01) (Figure 2D). After 24 h incubation, fossa cells showed a 13-fold increased expression of ADAMTS4 in response to 10 ng/mL IL1β (*p* < 0.01) (Figure 2D).

Six hours of IL-1β stimulation (10 ng/mL) also enhanced ADAMTS5 by 4-fold, but only in condylar cells (*p* < 0.01) (Figure 2E). After 24 h incubation with 10 ng/mL IL-1β, only fossa cells demonstrated enhanced ADAMTS5 gene expression (7-fold) in comparison to vehicle-treated cells (*p* < 0.017) (Figure 2E).

### 2.4. MMP-2 Activity Was Higher in Condyle Than Disc and Fossa Cells; MMP9 mRNA Upregulated in Condyle by IL-1β

**Figure 3:**
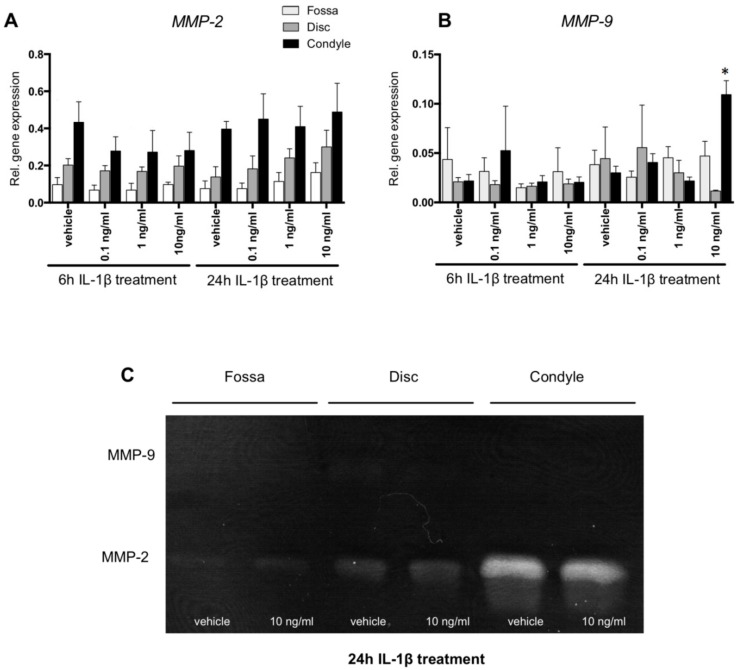
Matrix metalloproteinase (MMP)-2 and MMP-9 gene expression and activity. (**A**) IL-1β did not affect *MMP-2* expression by the cells from the fossa, disc, and condyle at any time point tested. (**B**) After 24 h of 10 ng/mL IL-1β incubation, the *MMP-9* gene expression of the disc and condyle cells were higher than that of the vehicle-treated samples. (**C**) Zymogram of the conditioned medium from fossa, disc, and condyle cells after 24 h of incubation with IL-1β. There was no MMP-9 activity detected. The condyle showed strong MMP-2 activity, but no effect of IL-1β was apparent. * Significant effect of treatment with IL-1β, relative to vehicle, *p* < 0.05. Results are shown from one out of three identical experimental replicates.

Six hours of IL-1β treatment did not affect MMP-9 gene expression in any of the TMJ-derived cell types (Figure 3B). After 24 h of stimulation with 10 ng/mL IL-1β, there was a 3-fold increase of MMP-9 gene expression by condyle cells (*p* < 0.01, Figure 3B). MMP-9 enzyme activity was undetectable by zymographic analysis of the conditioned medium of fossa, disc, and condyle cells, regardless of the IL-1β treatment (Figure 3C), suggesting that the mRNA for MMP-9 was not sufficiently converted into active protein. Though not statistically significant at the mRNA level (Figure 3A), MMP-2 enzyme activity appeared higher in condyle cells than in the disc and fossa (Figure 3C). IL-1β did not visibly affect the level of MMP-2 activity in any of the cells (Figure 3C). 

### 2.5. IL-1β Induced MMP-13 Expression by Condylar Cells Only

**Figure 4:**
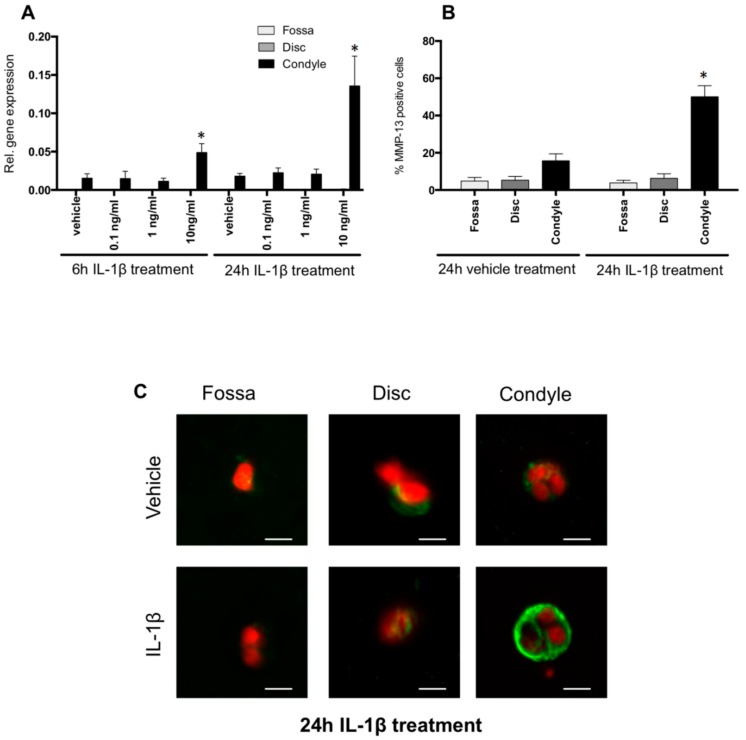
MMP-13 gene and protein expression. (**A**) MMP-13 gene expression by the cells from the fossa, disc, and condyle. IL-1β for 6 h and 24 h at 10 ng/mL increased MMP-13 expression in condyle cells in comparison to vehicle. (**B**) Number of MMP-13-positive cells. Condylar cells incubated with 10 ng/mL IL-1β for 24 h showed the highest number of MMP-13-positive cells. (**C**) Image of MMP-13-positive cells after 24 h of 10 ng/mL IL-1β treatment. The green label indicates the presence of MMP-13, and the nuclei are red. * Significant effect of treatment with IL-1β relative to vehicle treatment, *p* < 0.05. Scale bar represents 5 µm.

After 6 and 24 h of 10 ng/mL IL-1β stimulation, MMP-13 gene expression by cells of the condyle was up-regulated by 3.4- and 9-fold, respectively (*p* < 0.001 and *p* < 0.0001, respectively) (Figure 4A). MMP-13 gene expression was almost undetectable in the cells from the disc and fossa and remained low after IL-1β incubation (Figure 4A).

Next, we analyzed the number of cells expressing MMP-13 by immunostaining. Twenty-four hours of 10 ng/mL IL-1β incubation increased the percentage of MMP-13-positive condylar cells (3.5-fold increase, *p* < 0.001) (Figure 4B). The number of MMP-13-positive cells derived from the condyle compared to the fossa and disc was remarkably higher (Figure 4C).

### 2.6. Cyclic Tensile Strain Reduced IL-1β-Induced MMP-13 Expression

**Figure 5:**
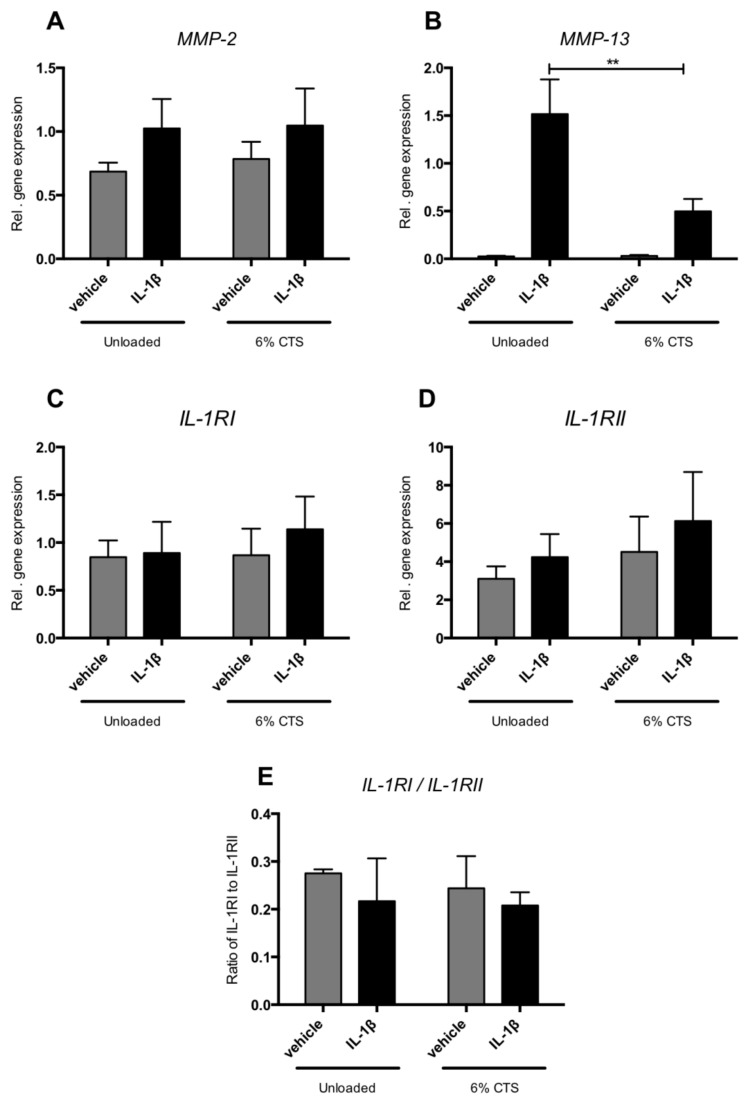
Mechanical strain reduces MMP-13 expression of condylar cells incubated with IL1-β. Gene expression of (**A**) MMP-13, (**B**) MMP-2, (**C**) IL1-RI, and (**D**) IL1-RII. (**E**) Ratio between IL-1RI and IL-1RII by condylar cells. Mechanical loading reduced IL-1β-induced gene expression of MMP-13. ** Significant effect of mechanical loading, *p* < 0.01.

Six percent cyclic tensile strain (CTS) reduced IL-1β-induced MMP-13 gene expression by 3-fold (*p* < 0.05) (Figure 5B). CTS neither affected expression of MMP-2, IL-12RI, IL-1RII nor the ratio of IL-1RI and IL-1RII in control condylar cells or in those incubated with IL-1β (Figure 5A,C,D).

## 3. Discussion

The TMJ is frequently affected in patients with chronic inflammation, which can result in permanent damage to the joint, especially in young patients. Since biological sampling of the TMJ of children for research purposes is unethical, the role of specific inflammatory factors in the degradation of the TMJ of young individuals remains elusive. In the present study, we made use of relatively young mice and juvenile porcine TMJs to investigate the effect of the inflammatory cytokine IL-1β on its three cartilaginous structures. Our findings strongly suggest that excess IL-1β induces degradation of TMJ cartilage. Young mice deficient for IL-1RA showed early histological signs of TMJ degradation, an effect preferentially found in the condyle. In culture, porcine cells isolated from the three cartilaginous structures expressed different catabolic enzymes in response to IL-1β, e.g., IL-1β at 10 ng/mL induced the expression of ADAMTS4 and ADAMTS5 by cells from the fossa, while cells isolated from the condyle responded to IL-1β with an increased expression of MMP-9, and MMP-13. Mechanical loading reduced MMP-13 expression in IL-1β-treated condylar fibrochondrocytes.

Horai et al. previously demonstrated that IL-RA^−/−^ mice developed spontaneous arthritis due to unopposed excess of IL-1 signaling. In this systemic arthritis model, between 5–20% of the front paws developed arthritis, which depended on, for instance, the microbiological status of the animal facility [20]. We used these mice to investigate whether an excess of IL-1 signaling could result in TMJ damage. We did indeed find some remarkable changes in the condyle. A high level of staining for proteoglycans was seen around the condyles and also in the fibrous areas of the condyle. This area normally does not contain proteoglycans. Condyles of the IL-RA^−/−^ mice showed, overall, more clustering of cells, more intense proteoglycan staining, and higher Mankin score in comparison to WT mice. Over-production of proteoglycans and cluster formation of chondrocytes may represent signs of local repair of articular cartilage, an indication of the onset of the cartilage degradation process. Proteoglycans are unlike collagen in a continuous turnover [20], therefore overshoot in matrix synthesis might occur more easily with proteoglycans. Other studies have also found an increased level of proteoglycans in the early phases of condyle cartilage degradation [21,22,23]. In these studies, at later stages, a gradual loss of proteoglycans occurred together with cleaving of collagen fibrils. This pattern of degeneration implies that there may be a common chain of molecular events underlying degeneration [21]. Further studies in older IL-1RA^−/−^ mice should indicate whether these mice will undergo loss of proteoglycans together with cleaving of the collagen fibrils in their TMJ by, for instance, MMP-13, which was upregulated in the porcine model. Taken together, our results with IL-1RA^−/−^ mice suggest that overactive IL-1β signaling induces damage in the fibrocartilage tissue of the condyle of the TMJ.

We assumed initially that the fossa and disc cells would not express the genes of the receptors related to IL-1β signaling, since these cartilage parts seemed to be unaffected in the inflamed joint of JIA patients [14]. However, we found that the cells from the fossa and disc expressed mRNA for these receptors, and cells from the fossa responded to IL-1β with an enhanced expression of ADAMTS4 and ADAMTS5. This shows that the receptors are present and functional in the fossa and disc, even though these structures are damaged to a lesser extent than the condyle in JIA patients. Increased ADAMTS5 expression in response to IL-1β in combination with lack of tissue damage was also observed in articular cartilage from knees of Sox9 knockout animals [24]. In addition, very limited numbers of proteoglycans are present in the fossa and disc. Therefore, with ADAMTS4 and ADAMTS5 being the catalytic enzymes that degrade proteoglycans, damage by these aggrecanases would be limited in comparison to the condyle. 

Condylar cells responded to IL-1β by increasing the expression of the catabolic enzymes ADAMTS5, MMP-9 and MMP-13. These cells also expressed constitutively active MMP-2. These enzymes are able to cleave the matrix proteins of the condylar cartilage. The aggrecanases ADSMTS5, MMP-13 and MMP-2 are capable of cleaving proteoglycans [25,26], and both MMP-13 and MMP-2 are able to unwind and cleave collagen fibrils [27]. The resulting fragments will form an excellent substrate for the gelatinase MMP-2. This enzyme is also able to cleave the pro-MMP-13, thereby activating this collagenase [28]. We found that IL-1β enhanced MMP-13 expression in cells isolated from the porcine condyle. The isolated cells constitute a mix of more fibroblast-like cells from the upper layer of the condyle and chondrocyte-like cells from the deeper layers. It is possible that only one of these subtypes of cells responds to IL-1β with increased MMP-13 expression. We found in a limited set of histological slides that MMP-13 protein was mostly expressed by chondrocyte-like cells of the deeper layers of mouse condyles (data not shown). It is thus possible that the response to IL-1β was most pronounced in the chondrocyte-like cells within our mix of isolated condyle cells. The importance of MMP-13 in cartilage degradation in arthritis was demonstrated in transgenic mice overexpressing MMP-13 [29] and elevated levels of MMP-13 were found in synovial fluid of arthritic patients [30]. Therefore, MMP-13 can be considered as one of the prime suspects in the degradation of condylar cartilage in JIA. Taken together, we found that IL-1β enhances the expression of catabolic enzymes by TMJ-derived cells, thereby possibly explaining cartilage damage as observed after overactive IL-1 signaling.

One limitation of this study is that we cannot be certain that histological changes indicative of degeneration in the condylar fibrocartilage of the TMJ of IL-1R^−/−^ mice can be attributed to MMP-13 over-expression. Studies using IL-1R^−/−^ mice treated with MMP-13 inhibitors could provide clarity, but such experiments were beyond the scope of the current study. In addition, our in vitro studies showing the effect of IL-1β on MMP-13 expression in condyle-derived fibrochondrocytes were performed with cells from pig TMJs but not mice, and species differences can occur. We have performed immunohistochemistry for MMP-13 on sections of mouse TMJs, but the resulting quality prevented accurate quantitative assessment, though roughly 60% of the condylar cells seemed positive in wildtype animals and nearly 100% in IL-RA knock-out mice (data not shown), which indicates that the effects of overactive IL-1β with regards to MMP-13 expression is similar between pig and mouse. Another limitation is the selection of only one mechanical loading regime of tensile forces, whereas compressive forces are also occurring in the moving jaw. 

Since MMP-13 plays an important role in many biological processes, including growth and development [31], inhibition of activity of this enzyme could have severe, undesirable side-effects in the children with JIA that are still growing. This important role of MMP-13 in many biological processes [31] requires a *direct* inhibition. Pharmaceutical intervention should therefore be based on tempering IL-1β’s destructive effects [32,33]. A potential non-invasive, non-pharmaceutical approach to inhibit inflammation-induced MMP-13 expression is exercise or physical therapy of inflamed joints. We found that 6% cyclic tensile strain exerted on the condylar cells significantly reduced the IL-1β-induced MMP-13 gene expression, similar to our previous finding that tensile strain exerted on condylar cells significantly reduced TNFα-induced MMP-13 gene expression [34]. These findings are in line with several other studies, in which the anti-catabolic capacity of cyclic strain was analyzed [34,35,36]. In our study, the cells maintained their pericellular matrix when they were embedded in an agarose gel, thereby allowing proper transmission of mechanical forces to the cells. The condylar cartilage undergoes considerable tensile forces due to compression and shear [37]. For this reason, we used 6% cyclic tensile strain. This percentage was calculated by using the following literature data. Deschner et al. used 20% of strain to stimulate rat disc cells [35], but Chain et al. calculated that the maximal tensile strain that the condyle cartilage would experience would be 3.7-fold lower than the disc [38]. Further in vivo studies are needed to assess whether 6% tensile strain is effective in downregulating catabolic enzymes induced by inflammation. 

In conclusion, overactive IL-1 signaling can induce changes in condyle cartilage metabolism indicative of degeneration, and cells from the three cartilaginous structures of the TMJ react to exposure to the inflammatory cytokine IL-1β, whereby the condyle seems particularly sensitive in terms of catabolic enzyme expression. This might explain why only the condyle is disproportionately degraded in children with JIA. MMP-13 induced by IL-1β might be a prime suspect in causing degradation of the condyle in JIA patients, and mechanical loading could inhibit expression. Future studies should confirm whether a direct link exists between JIA, IL-1β and MMP-13 over-expression, and whether controlled exercise can reduce MMP-13 expression in the condyle of the TMJ in vivo. These are important future steps with high clinical relevance because controlled physical exercise could provide a therapeutic intervention in children with JIA, potentially preventing serious effects of TMJ inflammation such as pain, dysfunction, and even malformations. Non-invasive studies, for instance using MRI, could be useful to monitor the effect of motion on the progression of JIA. 

## 4. Materials and Methods

### 4.1. Mice

IL-1RA-deficient (IL-1RA^−/−^) mice on a BALB/c background were kindly supplied by Martin Nicklin (Sheffield, UK). Wild-type control mice were 8–10 weeks old and were purchased from Charles River (Sulzfeld, Germany). Before the age of 12 weeks, mice were sacrificed, heads were dissected and fixed in 4% formaldehyde for 6–12 weeks. Ethical permission was obtained in July 2013 at the Radboud University Nijmegen, RU-DEC 2013-096.

### 4.2. Histological Analysis of Murine TMJ

Mouse heads (four mice per strain) were decalcified for 6 days in 10% formic acid and 10% sodium citrate solution. The heads were then dehydrated, embedded in paraffin, and 5 µm-thick sagittal sections were cut. Safranin O-fast green and hematoxylin and eosin staining were performed. Mouse TMJ cartilage was evaluated by a blinded observer based on pericellular staining, chondrocyte arrangement, and structural appearance of the articular cartilage, using a modified Mankin score [21] (Table 1). 

### 4.3. Cell Isolation and Culture 

Because mouse TMJs only contain few cells, all in vitro studies were performed with cells isolated from pig TMJs. Heads of Dutch Landrace pigs (*Sus scrofa*), with a body weight in the range of 70–80 kg and aged 6–8 months old, were obtained from a local abattoir (Westford, Gorinchem, The Netherlands). Approval by the Animal Ethics Committee of the VU University was not required as the animals were not sacrificed for the purpose of the experiment. Within 4 h after sacrifice, the entire articular cartilage of the fossa and condyle and the whole disc were dissected. The cells were isolated as previously described [39,40]. The medium containing the cells was mixed 1:1 with 6% ultrapure low melting point agarose (Invitrogen, Carlsbad, CA, USA) to a final concentration of 1 × 10^6^ cells/mL, 3% agarose, 1× DMEM supplemented with 50 µg/mL ascorbic acid (Merck, Darmstadt, Germany), 10% fetal bovine serum (FBS) (Thermo Fisher Scientific, Waltham, MA, USA), and 1% penicillin/streptomycin/fungizone (Invitrogen). The non-solidified gel was poured in a 2 mL syringe with a diameter of 8 mm of which the needle end was cut off, leaving a cylinder with an open needle-end. After gelation, the cell-gel construct was gently pressed out using the plunger and was cut into slices with a 2 mm thickness and transferred into a 24-wells plate. The 3D constructs were cultured for 6 days (Table 2) using a previously described protocol that is suitable for culturing chondrocytes [41]. On day 6, the cells were incubated with vehicle (PBS) or with 0.1, 1, or 10 ng/mL (7) recombinant porcine IL-1β (R&D Systems, Minneapolis, MN, USA) for 6 or 24 h.

For mechanical loading experiments, the cell-gel solution was poured on the silicone membrane of a Flexcell tissue train plate (Dunn Labortechnik, Asbach, Germany) on which two Velcro strips were glued. The Velcro strips ensured that the agarose cell-gel constructs would stick to the membrane of the tissue train plate. The rectangular shape of the cell-gel construct was confined by a 3D-printed mold to match the standard dimensions of gels on a tissue train plate. The cell-gel constructs were cultured as described in Table 2 for 6 days before performing tensile strain experiments. In the cyclic tensile strain experiments, the cells were incubated first for 12 h with IL-1β (10 ng/mL, R&D Systems), followed by 6% of sinusoidal mechanical strain at 0.5 Hz for 8 h, which was applied using the Flexcell system. Cells were post-incubated without strain for 24 h with IL-1β.

### 4.4. RNA Extraction and Real-Time Quantitative PCR

After 6 and 24 h of incubation with IL-1β, the cell-gel constructs were snap-frozen, and the RNA was extracted according to the protocol developed by Bougault and co-workers [41], cDNA was made using SuperScript^®^ VILO^TM^ cDNA Synthesis Kit according to the manufacturer’s instructions (Life Technologies, Carlsbad, CA, USA). Real-time PCR reactions were performed according to the manufacturer’s instructions in a LightCycler480^®^ (Roche Diagnostics, Switzerland). The sequences of the primer pairs are presented in Table 3. 

### 4.5. Zymography

MMP-2 and MMP-9 activities in the conditioned medium of IL-1β-treaded cells (10 ng/mL for 24 h) were analyzed by zymography. The medium was lyophilized and concentrated three times. Novex^®^ 10% Zymogram (Gelatin) Protein Gels, 1.0 mm, 15-wells (Life Technologies) were used for electrophoresis. After 1.5 h of electrophoresis, the gel was incubated for 30 min at room temperature in renaturing buffer (Life Technologies) with gentle agitation. The buffer was replaced with developing buffer (Life Technologies) for 30 min at room temperature and subsequently replaced for fresh buffer. After overnight incubation, the gels were washed with demi water and stained for 1 h with SimplyBlue SafeStain (Life Technologies). MMP-2 and MMP-9 activity was displayed as unstained bands.

### 4.6. Immunohistochemistry

Cell-gel constructs were fixed with formaldehyde and incubated for 2 h at room temperature with blocking buffer. Immunolocalization of MMP-13 was performed by using rabbit polyclonal anti-human MMP-13 (1:1000) (ab84594; Abcam, Cambridge, MA, USA) overnight at 4 °C. The secondary antibody alexa-555 goat anti-rabbit (1:2000 dilution) (A31630; Invitrogen) was incubated for 2 h at room temperature. Negative control staining was performed with Dako rabbit negative control (Dako, Glostrup, Denmark). Nuclei were stained with 4′,6-diamidino-2-phenylindole (DAPI). 

Six µm optical sections were made of the gels with an Axio ZoomV16 microscope (Zeiss, Munich, Germany). The micrographs were then superimposed, and the number of positive cells was counted. With this technique, we were able to scan through 200 µm gel and count the MMP-13-positive cells in these areas.

### 4.7. Statistical Analysis

Prism (GraphPad Software Inc., San Diego, CA, USA) was used for statistical analyses. Mean values and standard errors of the mean (SEM) were calculated and depicted in the figures throughout the manuscript. Differences in pericellular staining of extracellular matrix (proteoglycans), chondrocyte arrangement, and structural appearance of the articular cartilage between four wild-type and four IL-1RA^−/−^ mice were tested using the Mann–Whitney *U* test (*n* = 4). For each parameter measured, the null hypothesis was that cartilage damage-related changes scored equally in TMJs from WT and IL-1RA^−/−^ mice. Experiments with pig cells were performed at three separate occasions. Data obtained at one separate occasion were considered *n* = 1 (thus, total *n* = 3). At each separate occasion, a *new* cell pool (derived from three pig heads) per anatomical region was created. Cell pools derived from the condyle, fossa, and disc were kept separate, and all were treated with or without IL-1β. To determine whether IL-1β (0.1, 1, or 10 ng/mL) significantly affected gene expression of ADAMTS4, ADAMTS5, MMP-2, MMP-9 and MMP-13, as well as protein expression for MMP-13, and activity of MMP-2 and MMP-9, compared to vehicle, in TMJ-derived cell populations, Dunnett’s multiple comparison test was performed. Bonferoni correction was applied, as three tests were performed per parameter (one for fossa, one for disc and one for condyle) at the 6 h and at the 24 h time point, separately. At a *p* < 0.017, the null hypothesis, i.e., IL-1β (at either 0.1, 1, or 10 ng/mL) did not affect gene or protein expression of the catabolic factor of interest in pig TMJ cells, was rejected. To test the effect of mechanical strain on IL-1β-induced gene expression by pig condylar fibrochondrocytes, one-way ANOVA was performed. Differences were regarded significant at values of *p* < 0.05. 

## Figures and Tables

**Table 1:** Modified Mankin score.

**1) Pericellular Safranin O staining**	
a. Normal	0
b. Slightly enhanced	1
c. Intensely enhanced	2
**2) Background Safranin O staining**	
a. Normal	0
b. Slight decrease/increase	1
c. Severe decrease/increase	2
d. No staining	3
**3) Arrangement of Chondrocytes**	
a. Normal	0
b. Appearance of clustering	1
b. Hypocellularity	2
**4) Cartilage Structure**	
a. Normal	0
b. Fibrillation in superficial layer	1
c. Fibrillation beyond superficial layer	2
d. Missing articular cartilages	3

**Table 2:** Timetable of progressive substitution of serum for ITS in the cell-agarose construct.

	Fetal Bovine Serum (%)	ITS (%)	Ascorbic Acid (µg mL^−1^)	PSF (%)
Day 0	10	-	50	2
Day 1	5	-	50	2
Day 2	1	1	50	1
Day 4	-	1	50	1
Day 6	-	1	50	1

**Table 3:** Primers used for real-time PCR.

Genes	Primers	Primer Sequences ^a^
*YWHAZ* (reference gene)	Forward:	GATGAAGCCATTGCTGAAACTTG
	Reverse:	CTATTTGTGGGACAGCATGGA
*HPRT* (reference gene)	Forward:	GCTGACCTGCTGGATTACAT
	Reverse:	CTTGCGACCTTGACCATCT
*IL-1RI*	Forward:	CATGACTGCCCATTGTTGAG
	Reverse:	AGGGCAGAAGCCTAGGAAG
*IL-1RII*	Forward:	GTGCCTGTTGAGCCTCATT
	Reverse:	GGCCTTCATGGGCAAATGTCA
*ADAMTS4*	Forward:	CATCCTACGCCGGAAGAGTC
	Reverse:	GGATCACTAGCCGAGTCACCA
*ADAMTS5*	Forward:	GTGGAGGAGGAGTCAGTTTG
	Reverse:	TTCAGTGCCATCGGTCACCTT
*MMP-2*	Forward:	CCGTGGTGAGATCTTCTTCTTC
	Reverse:	GCGGTCAGTGGCTGGGGTA
*MMP-9*	Forward:	ACAGGCAGCTGGCAGAGGA
	Reverse:	GCCGGCAAGTCTTCCGAGTA
*MMP-13*	Forward:	GGAGCATGGCGACTTCTAC
	Reverse:	GAGTGCTCCAGGGTCCTT

^a^ Only primers with equal efficacy were used.
